# The Importance of Bacteriological Findings in Relation to the Treatment of Infected Teeth

**Published:** 1918-10

**Authors:** Montgomery La Roche


### THE IMPORTANCE OF BACTERIOLOGICAL FINDINGS IN RELATION TO THE TREATMENT OF INFECTED TEETH

BY MONTGOMERY LA ROCHE, D.D.S.

The greatest importance is attached to the teeth as a possible source of systemic disease. The dental profession and the world at large are having that fact pounded into them every minute by the great accumulation of evidence which is piling up day after day.

It is therefore bootless to go into the details of this evidence in the present article, but we must ever keep before us the prime importance of what we are doing in our daily work.

In dealing with dead teeth we must not be guided by the X-ray findings alone. There is no denying the fact that these leave much to be desired, however necessary to our work they may be. In fact, too much can not be said about the importance of good roentgenographic work in connection with the treatment of these teeth, although it must not be forgotten that we are dealing with microscopic not macroscopic beings, primarily bacteria. And unless they have been there a long while and their action while there has been of a certain character the result of their presence will not be exhibited in the roentgenogram. In any case the germs themselves will not be seen.

We are further confronted with the fact that after a certain amount of attempted sterilization of an infected root we are at a loss for proof of absolute sterility. We know through sad experience that lack of pain is no indicator. We know furthermore that the X-ray does not always indicate the presence or absence of microorganisms. Where then are we to turn for proof, for proof that is absolute and unquestioned, we must have. Otherwise we are not justified in treating these teeth.

Rhein claims that upon the injection of hydrogen peroxide into the root, the absence of bubbling indicates sterility. But is that *absolute proof?* Does that indicate the condition at and beyond the apex? He does not claim it does, and we must look further.

Up to the present time there has been developed nothing better for the purpose, so far as I have been able to ascertain, than the actual bacteriological findings, carried out in conjunction with the proper technic. In proceeding with the fact in mind that we are dealing with things invisible to the naked eye, we must not forget that there are germs constantly on our hands, in the air, on the bracket table, in our cabinets and in fact everywhere, and we must therefore be sure that everything that goes in that tooth is absolutely sterile. Sterilize the outside surfaces of the tooth, the adjacent parts of the dam and clamp, washing them off thoroughly with alcohol and iodin, being sure the alcohol has thoroughly evaporated before applying the iodin.

Open the pulp chamber carefully, using nitrate of silver to swab it out before opening the root or roots. Remove the debris in these roots as far as possible, using utmost care not to push any through the foramen and also observing strict sterility throughout, not touching the ends of the instruments with the fingers and using a little sulphuric acid or sodium-potassium. It is not desirable to open the roots and cleanse them at once if one wishes to ascertain the nature of the infection at the start. I find this to be an advantage in checking up later on in the treatment. This course is purely optional, however, and modifications are perfectly in order, especially where persistent pain is present.

Now we come to the crux of the whole subject. It is right here that the microscope and artificial media become necessary, and we are also confronted by a rather peculiar and puzzling fact, *viz.*: that it is a very difficult matter to get a culture from an infected root. I have placed broaches, platinum wires and loops into the canals and pulp chambers and obtained indifferent results, even when there was pus flowing from them. I have placed beef broth in the roots for a few moments and removed it under sterile conditions, with a little better results perhaps, but still unsatisfactory. I have placed sterile paper points in the roots, sealing them in for days, with very unsatisfactory results. I have therefore come to putting glucose broth in the roots, injecting it into them with a syringe, sealing sterile paper points in them, saturated with this broth, for forty-eight hours, and removing the paper points to a sterile glucose agar slant, which is incubated about twenty-four to forty-eight hours, and if there are germs there I get them. They can not resist this tempting treatment.

I hope I do not seem monotonous in constantly harping on sterility, but we as dentists have not been trained to the necessary precision in that direction. I should highly recommend a post graduate course in bacteriology as being not only instructive in itself, but as serving as a ground work for the understanding of the necessity for and meaning of absolute asepsis.

If we have followed out carefully the technic outlined above, we make our results absolutely sure, taking the newest, most far-reaching and most important developments in modern medical and dental science, the actual elimination of foci of infection, out of the realms of uncertainty and guess work and placing it in the bright light of scientific knowledge. We can not go wrong and therefore we must not continue to do as we have been doing in the past. We must not neglect the open door of opportunity at our elbow, but we must eliminate all doubts in our work. Our patient's health is at stake and when we once realize the importance of our work, no task can be too exacting, no care too great.—*The Journal of Allied Dental Societies*, June. 1918.

